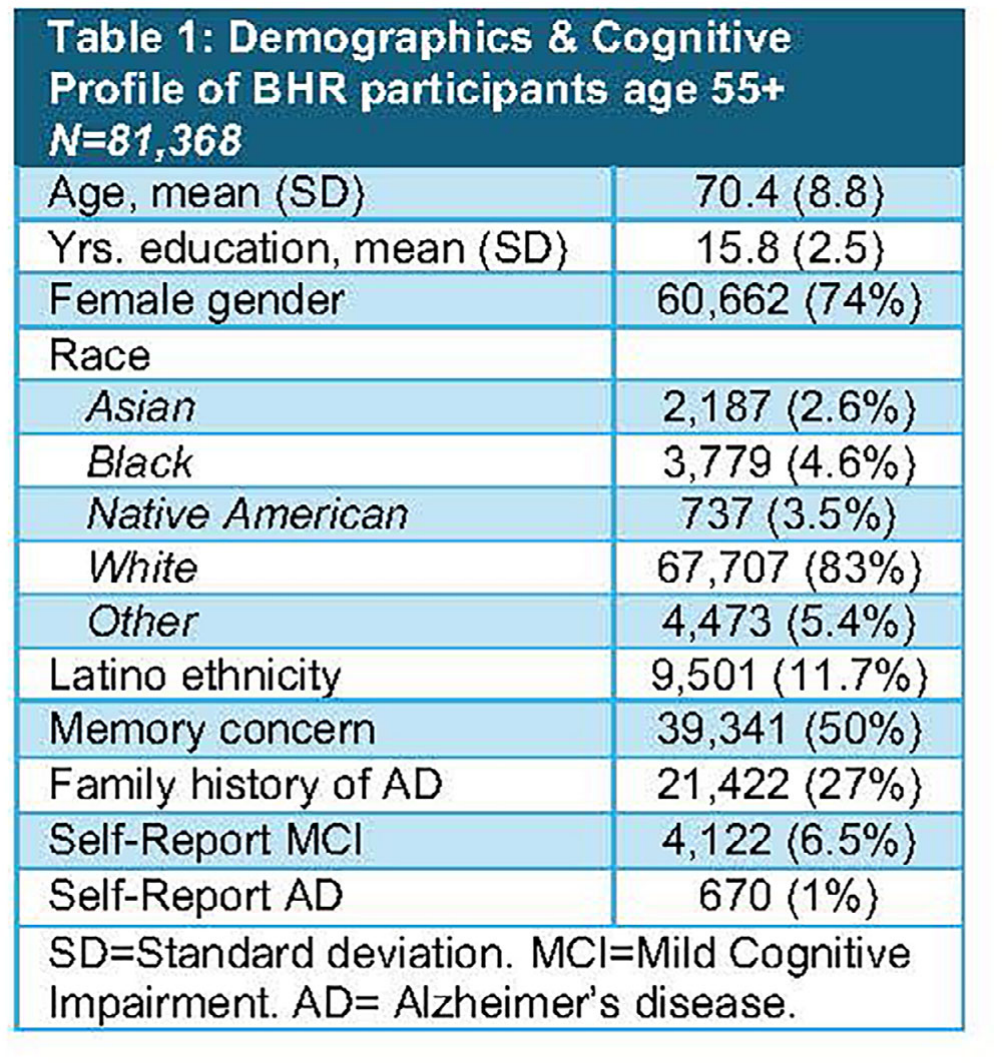# The Brain Health Registry: Facilitating AD and aging clinical research through participant referral to clinical studies

**DOI:** 10.1002/alz70860_098468

**Published:** 2025-12-23

**Authors:** Rachel L. Nosheny, Miriam T. Ashford, Joseph Eichenbaum, Monica R. Camacho, Derek Flenniken, Juliet Fockler, Winnie Kwang, Diana Truran‐Sacrey, Scott R. Mackin, Michael S. W. Weiner

**Affiliations:** ^1^ University of California, San Francisco, San Francisco, CA, USA; ^2^ San Francisco Veterans Affairs Medical Center, San Francisco, CA, USA; ^3^ Northern California Institute for Research and Education (NCIRE), San Francisco, CA, USA

## Abstract

**Background:**

Recruitment of older adults for clinical Alzheimer's Disease (AD) and aging observational studies and trials is expensive, time consuming, and causes study delays. Many studies have inadequate recruitment. Internet‐based registries are an efficient, scalable approach to help enroll participants into AD research. The Brain Health Registry (BHR) is an online registry and observational cohort that addresses this critical need by referring participants to AD and aging observational studies and trials.

**Methods:**

BHR includes an engaging, online platform for registration, consent, and assessment. Participants enroll through the BHR website, sign online informed consent, and complete digital questionnaires and cognitive tests at 6‐month intervals. All assessments are completed remotely, unsupervised, on one's own device. Questionnaires include demographics, determinants of health, subjective cognitive decline, head injury, mood, and medical history. Cognitive tests include the Cambridge Cognition Paired Associates Learning test. BHR participants who opt in to learning about future research opportunities can be referred to additional studies, including collaborator studies and sub‐studies led by BHR investigators. The BHR database is used to identify participants who meet study inclusion and exclusion criteria. Eligible participants receive automated emails inviting them to the study and explaining next steps. BHR demographic, cognitive, and health data is often used to screen and prioritize participants for referral. Collaborators can manage their referrals online through an Investigator Portal within BHR.

**Results:**

BHR includes >104,000 participants and >10,000 study partners, including >81,000 participants age 55+ (Table 1), and >20,000 from minoritized ethnocultural groups. Over 90% of all BHR participants opt in to learning about future research opportunities. Since 2014, BHR has completed >489,000 invitations to additional studies. From those, >42,000 participants have enrolled in at least one of 35 collaborator studies. This includes in‐clinic observational studies, online studies, and clinical trials.

**Conclusions:**

The results support use of BHR to facilitate efficient recruitment into AD and aging clinical research studies. Prescreening using BHR data can be used to reduce burden of in‐clinic screening. Planned improvements to the referral process include further automation of participant selection and communications to facilitate recruitment of participants across different demographic strata (e.g., age, education, ethnocultural identity).